# Effect of Substance Abuse on Suicidal Behaviors Among People Living With HIV: 11-Year Cohort Study

**DOI:** 10.1097/jnr.0000000000000730

**Published:** 2026-03-12

**Authors:** Yi-Tseng TSAI, Sriyani Padmalatha Konara MUDIYANSELAGE, Tzu-Jung CHUANG, Chung-Yi LI, Shu-Sen CHANG, Mu-Hong CHEN, Nai-Ying KO

**Affiliations:** 1 School of Nursing, China Medical University, Taichung, Taiwan; 2 Department of Nursing, College of Medicine, National Cheng Kung University, Tainan, Taiwan; 3 Operating Room Department, The National Hospital of Sri Lanka, Colombo, Sri Lanka; 4 Institute of Behavioral Medicine, College of Medicine, National Cheng Kung University, Tainan, Taiwan; 5 Department of Public Health, College of Medicine, National Cheng Kung University, Tainan, Taiwan; 6 Institute of Health Behaviors and Community Sciences, College of Public Health, National Taiwan University, Taipei, Taiwan; 7 Department of Psychiatry, Taipei Veterans General Hospital, Taipei, Taiwan

**Keywords:** depression, HIV, suicide, substance abuse, sleep disturbances

## Abstract

**Background:**

The suicide rate among individuals with substance abuse disorders is three times higher than in the general population, with nearly half of people living with human immunodeficiency virus (PLHIV) experiencing substance abuse.

**Purpose:**

This study was implemented using 11 years of official data to identify the risk factors of suicide and their association with substance abuse among PLHIV.

**Methods:**

A nationwide cohort study was conducted in Taiwan using statistics from the national HIV database dating from January 1, 2005, to December 31, 2016. PLHIV aged 15 and older were identified using recorded diagnoses; medical treatments were identified using unique identification codes (ICD). Cox proportional hazard models and mediation analysis were used to estimate the association between substance abuse and suicide risk.

**Results:**

Fifty-nine suicide events were reported among the 4,016 PLHIV tracked in the study data, correlating with a suicide incidence rate of 743.91 per 100,000 person-years among substance abusers (*n*=1,287). Two factors, including substance abuse and low income, were found to significantly increase the hazard ratio (HR) of suicide (HR=1.93, 95% CI=[1.12, 3.33], *p*=.0174 and HR=5.15, 95% CI=[1.58, 16.86], *p*=.0067, respectively). Also, sleep disturbances were shown to mediate 19.1% of the effect between substance abuse and suicide. Conversely, depression did not exhibit a significant mediating role.

**Conclusions:**

The findings indicate that substance abusers face a nearly twofold higher risk of suicide than nonusers, with significant socioeconomic disparities evident. Moreover, sleep disturbances was identified as a critical mediator in the relationship between substance abuse and suicide. These findings provide valuable insights to help improve clinical practices and policy development efforts aimed at preventing suicidal behaviors and improving the well-being of PLHIV struggling with substance abuse globally.

## Introduction

Suicide, a significant global public health concern, causes ~800,000 deaths annually, which translates to approximately one death every 40 seconds (WHO, 2023). People living with human immunodeficiency virus (PLHIV) are particularly vulnerable, facing a threefold higher risk of suicidal behavior than the general population (Stubbs et al., 2016; Wallace et al., 2020). Substance abuse involving alcohol, illicit drugs such as cocaine and opioids, and other similar substances is common among high-risk groups for HIV infection, affecting up to 48% of substance abuse individuals living with HIV (Hartzler et al., 2017; Hitch et al., 2019; Kolla et al., 2020). In the post-highly active antiretroviral therapy (HAART) era, between 1997 to 2020, the prevalence of suicide attempts among PLHIV with substance abuse ranged from 24.8% to 35.3% (Bantjes et al., 2017; Byrne et al., 2024).

An estimated one-third of individuals living with HIV/AIDS who also have substance abuse disorders have reported a history of suicide attempts (Walter & Petry, 2016). The social stigma associated with HIV-positive status, together with the daily administration of multiple medications and their recurrent adverse effects, creates a potentially traumatic experience that increases the risk of sleep disturbances and depression. Previous studies have shown a positive correlation, as indicated by univariate analyses, between posttraumatic stress disorder, depressive symptoms, sleep disturbances, and substance abuse (including cocaine, opioids, marijuana, psychoactive agents, and multiple drugs) and engagement in suicidal behaviors (Tsai et al., 2024). There are interconnected relationships between substance abuse, sleep disturbances, and depression, all of which contribute to suicidal behavior risk (Fang et al., 2019). A comprehensive review of data from the United Kingdom revealed significant disparities in the prevalence rates of depression (50%–58% among PLHIV compared to 27% in the general population), sleep disturbances (61% among PLHIV compared to 10% in the general population), and suicidal ideation (31% among PLHIV compared to 1% in the general population; Chaponda et al., 2018). Moreover, the literature provides extensive support for associations between substance abuse, sleep disorders, and depression, respectively, and suicide (Mudiyanselage et al., 2025; Tsai et al., 2022). Furthermore, the findings of previous studies indicate that the presence of depression and sleep disturbances increases susceptibility to developing substance abuse (Fang et al., 2019).

Major depressive episodes often co-occur with sleep disturbances and substance abuse, pointing to associations between sleep disturbances and depression and suicide (Lu et al., 2021; McCall, 2015). Individuals living with HIV who experience depression and substance abuse commonly exhibit a notably high prevalence of sleep disturbances (Lee et al., 2024; Taibi, 2013). In the general population, suicide rates among people with sleep disturbances range from 10.5% to 30.9% (Fan et al., 2019). Sleep disturbances and depression are common comorbidities among PLHIV and are known to exacerbate suicidal tendencies (Chaponda et al., 2018). However, their roles as mediators of suicidal behavior remain inadequately studied, particularly in long-term cohort analyses. While previous studies have established a link between substance abuse and suicide, robust evidence of the mediating roles of depression and sleep disturbances in this population, particularly in non-Western settings, is lacking (Harris et al., 2020). Taiwan’s comprehensive National Health Insurance (NHI) system provides a unique opportunity to study a large and representative cohort of PLHIV using reliable longitudinal data. In this study, an 11-year cohort from Taiwan is used to explore not only the association between substance abuse and suicide but also the mediating roles of depression and sleep disturbances.

This study aims to provide evidence for clinical practice and policy development to mitigate the risk of suicide among PLHIV with substance abuse. In addressing critical mediators such as sleep disturbances, the findings highlight potential areas for targeted interventions.

## Methods

### Data Source

Patient records with HIV diagnoses between January 1, 2006, and December 31, 2012, were obtained from the National Health Disease Database of HIV/AIDS. In Taiwan, health care providers are required to report patient information within 24 hours of HIV/AIDS diagnosis, resulting in the near-100% enrollment of Taiwan’s HIV population in this database. Patients aged 15 years and older at the time of HIV diagnosis, with more than three outpatient visit records for HIV treatment, were included in the analysis. The HIV/AIDS database was linked to the Registry for Beneficiaries using unique identification codes (ICD).

The National Health Insurance (NHI) database is a comprehensive repository of medical claim data, housing the medical records and health care procedures of beneficiaries in Taiwan. Effectively, all of Taiwan’s population (~99.99%) is enrolled in the NHI program (Hsieh et al., 2019). This cohort data analysis was conducted using the NHI database, the Taiwan Death Registry, and the HIV/AIDS database and covered the period January 1, 2005, to December 31, 2016. The multiple causes of death data were sourced from the Taiwan Death Registry of the Ministry of Health and Welfare Statistics Department. The Death Registry data used included patient demographic and disease information such as the deceased’s ID number, gender, age, place of residence, occupation, marital status, date of birth, and details regarding the time and place of death.

### Sample

All individuals with a record of making three or more outpatient visits to ambulatory care centers for the pharmacological management of primary substance abuse disorder from their date of HIV diagnosis until December 30, 2016, were included in this analysis. The enrollment date for each patient with substance abuse was treated as their date of diagnosis. The ICD-9-CM codes for substance abuse disorder (*n*=1,485) were included in Table 1. Three measures, that is, age, gender, and duration of substance abuse, were used to compare the control and HIV/AIDS groups. Participants in the control group were living without a confirmed record of substance abuse. The participants were followed from their date of substance abuse diagnosis (DOD) to either their date of suicide recorded in the NHI database or the last day of 2016. Patient age was calculated as the difference between the DOD date and the date of birth. Records were excluded if they contained missing demographic information, if individuals had a documented substance use disorder within 1 year before the HIV diagnosis date, if depression or sleep disturbance was diagnosed within 1 year before enrollment, or if a suicide attempt occurred during the 3-year follow-up period after enrollment (Tsai et al., 2024).

**Table 1:** Characteristics of PLHIV With/Without Substance Abuse

Characteristic	Overall (*n*=4,016)	HIV With Substance Use (*n*=1,287)	HIV Without Substance Use (*n*=2,729)	*p*
	*n* (%)	*n* (%)	*n* (%)	
Gender				.2259
Female	278 (6.92)	80 (6.22)	198 (7.26)	
Male	3,738 (93.08)	1,207 (93.78)	2,531 (92.74)	
Age (years; mean and *SD*)	40.33±8.43	40.90±8.40	40.06±8.43	.0030
≥50	571 (14.22)	202 (15.70)	369 (13.52)	.0427
40–49	1,292 (32.17)	428 (33.26)	864 (31.66)	
30–39	1,775 (44.20)	554 (43.05)	1,221 (44.74)	
≥15–29	378 (9.41)	103 (8.00)	275 (10.08)	
Premium-based monthly salary (NT $)				<.0001
≥24,000	1,138 (28.34)	119 (9.25)	1,019 (37.34)	
<24,000	2,878 (71.66)	1,168 (90.75)	1,710 (62.66)	
Level of urbanization				<.0001
High	1,866 (46.46)	471 (36.60)	1,395 (51.12)	
Medium	1,284 (31.97)	475 (36.91)	809 (29.64)	
Low	866 (21.56)	341 (26.50)	525 (19.24)	
Charlson Comorbidity Index (HIV not included)				<.0001
0	2,591 (64.52)	663 (51.52)	1,928 (70.65)	
≥1	1,425 (35.48)	624 (48.48)	801 (29.35)	
Marital status				<.0001
Marriage/cohabitation	365 (9.09)	117 (9.09)	248 (9.09)	
Unmarried	2,842 (70.77)	774 (60.14)	2,068 (75.78)	
Separated/divorced/widowed/unknown	809 (20.14)	396 (30.77)	413 (15.13)	
Employment status				<.0001
Employment	1,920 (47.81)	397 (30.85)	1,523 (55.81)	
Unemployment	1,433 (35.68)	672 (52.21)	761 (27.89)	
Students/ unknown/ others	663 (16.51)	218 (16.94)	445 (16.31)	
Comorbidities				
Sleep disturbance	458 (11.40)	244 (18.96)	214 (7.84)	<.0001
Anxiety	375 (9.34)	203 (15.77)	172 (6.30)	<.0001
Dementia	46 (1.15)	30 (2.33)	16 (0.59)	<.0001
Mental disorders	1,402 (34.91)	1,088 (84.54)	314 (11.51)	<.0001
Psychiatric disorders	249 (6.20)	167 (12.98)	82 (3.00)	<.0001
Neurological disorders	133 (3.31)	73 (5.67)	60 (2.20)	<.0001
Opportunistic infections	328 (8.17)	47 (3.65)	281 (10.30)	<.0001
Depression	265 (6.60)	175 (13.60)	90 (3.30)	<.0001
Receipt of HAART therapy				<.0001
Non-HAART users	3,694 (91.98)	1,233 (95.80)	2,461 (90.18)	
Regular HAART users (≥0.5 years)	322 (8.02)	54 (4.20)	268 (9.82)	
HAART adherence				<.0001
Adherence ≥85%	428 (10.66)	79 (6.14)	349 (12.79)	
Adherence <85%	122 (3.04)	36 (2.80)	86 (3.15)	
Never used HAART	3,466 (86.30)	1,172 (91.06)	2,294 (84.06)	
Frequency of emergency visits (per year)				<.0001
0	2,338 (58.22)	675 (52.45)	1,663 (60.94)	
≥1	1,678 (41.78)	612 (47.55)	1,066 (39.06)	
Follow-up time (mean and *SD*)	3.51±1.66	3.55±1.66	3.49±1.66	.3127
Median [Q1, Q3]	3.08 [2.38, 4.87]	3.08 [2.39, 4.96]	3.08 [2.38, 4.85]	

*Note.* To prevent time bias, the research population will be tracked for 3 years, so this part presents the situation between the enrollment date (diagnosis of substance abuse) and the enrollment date + 3 years. PLHIV = people living with HIV; HAART = highly active antiretroviral therapy.

### Ethical Approval

This study was approved as exempt by the institutional review board of a hospital in southern Taiwan (TMANH112-REC037) because anonymized databases were used. Access to Taiwan’s National Health Insurance Research Database was approved by the Ministry of Health and Welfare Review Committee. All data were handled in accordance with strict confidentiality and data protection regulations during data abstraction and analysis.

### Outcome

The outcome variables considered in this study included suicide behaviors and completed suicides due to substance abuse among PLHIV. All outcome data were retrieved using the ICD-9-CM and ICD-10-CM from the inpatient expenditures records in the NHI database and multiple causes of death data from the Death Registry. Cases in which substance abuse was observed were treated as prospective predictors of both attempted and completed suicides. The median duration of follow-up for these cases ranged from 2 to 10 years (Liu et al., 2020). To investigate the incidence of suicide, we analyzed data from PLHIV who were followed for a minimum of 3 years and had experienced an initial suicide attempt or completed suicide.

### Confounding Factors

Demographic variables collected in this study included gender, age, income, HIV transmission route, marital status, and employment status. Income was determined based on insurance payments, which relate to the patient’s income, and was categorized as either 0 to 23,999 NTD or more than 24,000 NTD. Comorbidities considered in this study included opportunistic infections, psychiatric disorders, and health care adherence. For patients with substance use disorders, the enrollment date was defined as the date of diagnosis, identified using International Classification of Diseases, Ninth and Tenth Revision (ICD-9-CM and ICD-10-CM) codes in accordance with established case ascertainment methodology (Tsai et al., 2024; Tables 1 and 2 for ICD details). Urbanization level was categorized into three groups—high (metropolitan cities), medium (small towns and suburban areas), and low (rural areas)—and was used as a proxy for socioeconomic status to examine its influence on the association between substance abuse and suicide risk among people living with HIV (PLHIV; Tsai et al., 2024).

**Table 2:** Overall and Covariate-Specific Incidence Rate of Suicide Among the PLHIV Cohort (N =14,107)

Characteristic	Person Year	No. of Events	Suicide Rate/per 100,000 Person-Years (95% CI)	*p*
Overall	14,107	59	418.23 [318.37, 539.48]	
Group				<.0001
With substance use	4,570	34	743.91 [515.18, 1039.54]	
Without substance use	9,537	25	262.15 [169.65, 386.98]	
Gender				<.0001
Female	997	6	601.90 [220.89, 1310.07]	
Male	13,110	53	404.26 [302.82, 528.79]	
Age (years)				<.0001
≥50	1,827	10	547.23 [262.42, 1006.38]	
40–49	4,249	18	423.64 [251.08, 669.54]	
30–39	6,319	26	411.47 [268.78, 602.89]	
≥15–29	1,712	5	292.05 [94.83, 681.54]	
Premium-based monthly salary (NT$)				<.0001
≥24,000	3,912	3	76.68 [15.81, 224.10]	
<24,000	10,195	56	549.29 [414.93, 713.30]	
Level of urbanization				<.0001
High	6,657	21	315.47 [195.28, 482.23]	
Medium	4,590	22	479.30 [300.38, 725.67]	
Low	2,860	16	559.35 [319.72, 908.35]	
Medical comorbidities per the Charlson Comorbidity Index (exclude HIV=6 points)				<.0001
0	9,375	36	383.99 [268.94, 531.60]	
≥1	4,732	23	486.06 [308.12, 729.34]	
Marital status				<.0001
Marriage/cohabitation	1,212	6	494.99 [181.65, 1077.38]	
Unmarried	10,075	41	406.96 [292.04, 552.09]	
Separated/divorced/widowed/unknown	2,820	12	425.49 [219.86, 743.25]	
Employment status				<.0001
Employment	6,801	20	294.06 [179.62, 454.15]	
Unemployment	4,898	28	571.61 [379.83, 826.13]	
Students/unknown/others	2,408	11	456.94 [228.10, 817.59]	
Comorbidities				
Sleep disturbance	1,457	16	1,097.96 [627.58, 1783.02]	<.0001
Anxiety	1,219	10	820.02 [393.23, 1508.04]	<.0001
Mental disorders	4,692	33	703.29 [484.11, 987.68]	<.0001
Psychiatric disorders	778	6	771.41 [283.10, 1679.04]	<.0001
Opportunistic infections	1,071	6	560.11 [205.55, 1219.13]	<.0001
Depression	902	12	1,330.00 [687.23, 2323.24]	<.0001
Receipt of HAART therapy				<.0001
Non-HAART users	13,302	56	420.98 [318.01, 546.68]	
Regular HAART user (≥0.5 years)	805	3	372.67 [76.85, 1089.10]	
HAART adherence				<.0001
Adherence ≥85%	1,040	4	384.65 [104.80, 984.85]	
Adherence <85%	250	2	801.14 [97.02, 2893.98]	
Never used HAART	12,817	53	413.49 [309.74, 540.86]	
Frequency of emergency visits (per year)				<.0001
0	8,413	30	356.59 [240.59, 509.06]	
≥1	5,694	29	509.29 [341.08, 731.43]	

*Note.* Suicide rate includes suicide attempts and deaths by suicide. PLHIV = people living with HIV; HAART = highly active antiretroviral therapy.

The level of health care adherence was determined based on clinic visit attendance for infectious diseases, receipt of HAART, and adherence to HAART medication. Physician visits were defined as having at least two clinical visits per year, with an interval of more than 180 days between the two visits. Patients receiving HAART were classified into two groups: non-HAART users and regular HAART users (≥0.5 years; Tsai et al., 2024). Based on the prescription refill records of each individual, adherence to HAART was categorized using the medication possession ratio (MPR), with a threshold of MPR ≥85% or <85%. MPR was calculated as the number of days for which medications were refilled divided by the total number of days from the enrollment date of HIV diagnosis (with/without substance abuse) plus 3 years (DOD date) to the end of the observation period (Tsai et al., 2024).

### Statistical Analysis

The suicide incidence rates were calculated separately for each variable using a stratified approach. A formula involving the division of the number of cases by the total person-years of observation for the exposed population was used as follows: ([number of suicide cases during the study period]/[time each person is observed in years after the index date, totaled for all persons])×100,000. This allowed the incidence per 100,000 person-years to be calculated for each specific age group. The age- and gender-specific annual rates were stratified into four groups: ≥15–29 years, 30–39 years, 40–49 years, and ≥50 years. Assuming a Poisson distribution of cases, 95% confidence intervals (CIs) were calculated. To evaluate the independent effects of substance use on suicide risk, the Cox proportional hazards model incorporating the time elapsed since the date of diagnosis of HIV with/without substance abuse (DOD date) was used to estimate hazard ratios and their corresponding 95% CI and logistic regression on the survival function against time. This model further included an interaction term between the covariates and the time (Tsai et al., 2024).

**Figure 1:**
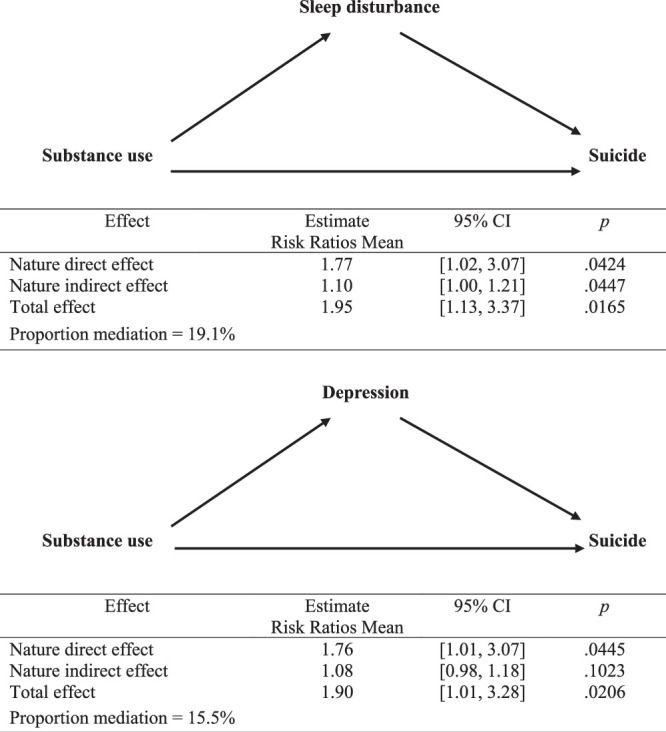
Mediation Analysis of Substance Use for, Respectively, Suicide by Sleep Disturbance and Substance Use for Suicide by Depression

The data were adjusted for demographic, comorbidities, environmental, and health care behavior factors that contributed to the model. Urbanization level was adjusted to account for the urban-rural difference in accessibility to medical care in Taiwan (Tsai et al., 2024). Categorical and continuous variables were analyzed using *t* tests or χ^2^ tests to identify any significant differences. To estimate the risk of suicide, the direct and indirect effects of sleep disturbance and depression mediated by substance abuse were assessed using a causal mediation analysis approach, following the formula proposed by VanderWeele (2011; Figure 1). Statistical analyses were performed using SAS statistical software (version 9.4; SAS Institute, Cary, NC, USA). A significance level of <.05 was applied to all tests.

## Results

### Demographic Characteristics

A total of 14,549 patients with an HIV diagnosis were obtained from the HIV database for the period 2006–2012. Of this number, 1,688 were excluded for missing information (431), age <15 years old at the time of HIV diagnosis (17), substance abuse within 1 year before the HIV diagnosis date (324), enrollment date occurring after 2012 (725), or a follow-up duration of <3 years from the enrollment date (191).

A total of 1,914 were coded with a history of substance abuse. Through the match sampling method, 1–2 control subjects were identified based on gender, age, and year of sleep disturbance diagnosis. A total of 1,485 PLHIV with substance abuse and 2,970 (77.5%) without substance abuse after HIV diagnosis were found. In the final 4,016 HIV cohort population, 1,287 had a substance abuse diagnosis (34 suicides: 20 suicide attempts and 14 deaths by suicide) and 2,729 did not have a substance abuse diagnosis (25 suicides: 11 suicide attempts and 14 deaths by suicide) or completed suicides for the second outcome (Figure 1).

The 4,016 patients included in this study were mostly male (93.08% vs. 6.92% female). The average age at baseline was 40.33 years. The average age at the time of diagnosis for those with substance abuse was 40.90 years. Among PLHIV without substance abuse, the primary diagnosis was identified in 2,531 males and 198 females. The average follow-up time for the HIV population was 3.51 years. Compared with PLHIV without substance abuse, those with substance abuse were younger, more likely to have lower income (<NTD 24,000) or be unemployed, more likely to reside in medium- or high-urbanization areas, and had a higher comorbidity burden. The combined prevalence of mental disorders in this group was as high as 84.54%. In addition, individuals with substance abuse were less likely to receive highly active antiretroviral therapy (HAART). In addition, the substance abuse group had a lower frequency of emergency department visits than the non–substance abuse group (Table 1).

### Suicide Incidence Among PLHIV

The overall incidence of suicide among PLHIV was found to be 418.23 per 100,000 person-years, with 59 events occurring over 14,107 person-years. When comparing groups, those with substance abuse had a significantly higher suicide rate of 743.91 per 100,000 person-years across 4,570 person-years, while those without substance abuse had a rate of 262.15 per 100,000 person-years over 9,537 person-years. The gender-specific data show females had a suicide rate of 601.90 per 100,000 person-years with 6 events across 997 person-years, while males had a rate of 404.26 per 100,000 person-years with 53 events over 13,110 person-years (Table 2).

### Suicide Risk Factors Associated With Substance Abuse Among PLHIV

The crude hazard ratio for suicide in the substance abuse group was significantly higher at 2.84, which remained elevated at 1.93 after adjustment. Males had a lower, though not statistically significant, hazard ratio of 0.68 compared to females. Age did not significantly affect the hazard ratios, with those aged 15–29 years having the lowest, albeit not significant, hazard ratio of 0.46. Income significantly influenced suicide risk, with those earning <24,000 NTD having a much higher adjusted hazard ratio of 5.15. The hazard ratios were also higher for those with lower levels of urbanization, though not statistically significant. Employment status also influenced risk, with unemployed individuals having a crude hazard ratio of 1.95, but this association was not significant after adjustment. Comorbidities such as anxiety and psychiatric disorders were associated with increased crude hazard ratios of 2.18 and 1.96, respectively, but these associations were not significant after adjustment. No significant associations were observed in terms of receipt or adherence to HAART therapy, nor for the frequency of emergency department visits (Table 3).

**Table 3:** Hazard Ratios of Suicide in PLHIV With Substance Use Compared With Control Participants From the HIV Cohort

Characteristic	Crude			Adjusted		
	Regression Coefficient (*SE*)	Hazard Ratio/95% CI	*p*	Regression Coefficient (*SE*)	Hazard Ratio/95% CI	*p*
Case						
Without substance use		Reference			Reference	
With substance use	1.04 (0.26)	2.84 [1.69, 4.75]	<.0001	0.66 (0.28)	1.93 [1.12, 3.33]	.0174
Gender						
Female		Reference				
Male	−0.38 (0.43)	0.68 [.29, 1.59]	.3763			
Age (y)						
≥50		Reference				
40–49	−0.27 (0.39)	0.76 [.35, 1.65]	.4939			
30–39	−0.33 (0.37)	0.72 [.35, 1.50]	.3827			
≥15–29	−0.78 (0.55)	0.46 [.16, 1.36]	.1616			
Premium-based monthly salary (NT$)						
≥24,000		Reference			Reference	
<24,000	1.94 (0.59)	6.99 [2.19, 22.34]	.0010	1.64 (0.60)	5.15 [1.58, 16.86]	.0067
Level of urbanization						
High		Reference				
Medium	0.41 (0.31)	1.51 [.83, 2.75]	.1769			
Low	0.59 (0.33)	1.80 [.94, 3.46]	.0759			
Medical comorbidities per the Charlson Comorbidity Index (exclude HIV=6 points)						
0		Reference				
≥1	0.27 (0.27)	1.31 [.77, 2.20]	.3203			
Marital status						
Marriage/cohabitation		Reference				
Unmarried	−0.20 (0.44)	0.82 [.35, 1.92]	.6401			
Separated/divorced/widowed/unknown	−0.16 (0.50)	0.85 [.32, 2.27]	.7481			
Employment status						
Employment		Reference			Reference	
Unemployment	0.67 (0.29)	1.95 [1.10, 3.45]	.0231	0.24 (0.30)	1.27 [.71, 2.30]	.4229
Students/unknown/others	0.44 (0.38)	1.55 [.74, 3.23]	.2447	0.21 (0.38)	1.23 [.59, 2.59]	.5830
Comorbidities						
Anxiety	0.78 (0.35)	2.18 [1.10, 4.30]	.0248	0.60 (0.35)	1.82 [.91, 3.62]	.0907
Psychiatric disorders	0.68 (0.43)	1.96 [.84, 4.57]	.1172			
Neurological disorders	0.18 (0.72)	1.20 [.29, 4.92]	.7983			
Opportunistic infections	0.33 (0.43)	1.39 [.60, 3.24]	.4438			
Receipt of HAART therapy						
Non-HAART users		Reference				
Regular HAART user (≥0.5 years)	0.01 (0.60)	1.01 [.31, 3.26]	.9885			
HAART adherence						
Adherence ≥85%		Reference				
Adherence <85%	0.76 (0.87)	2.14 [.39, 11.67]	.3814			
Never used HAART	−0.07 (0.53)	0.94 [.34, 2.62]	.9010			
Frequency of emergency visits (per year)						
0		Reference				
≥1	0.36 (0.26)	1.43 [.86, 2.39]	.1665			

*Note.* The number of suicides is overly small. Thus, adjusted analysis employing Crude with significance (*p*<.05) was used. PLHIV = people living with HIV; HAART = highly active antiretroviral therapy.

### Medication Analysis for a History of Substance Abuse and Depression

The mediation analysis of the relationship between substance abuse and suicide among PLHIV, with sleep disturbance and depression as mediators, is illustrated in Figure 1. The analysis reveals sleep disturbances mediated 19.1% of the effect of substance abuse on suicide, with a natural indirect effect hazard ratio of 1.10 and a natural direct effect hazard ratio of 1.77, resulting in a total effect hazard ratio of 1.95. Similarly, depression mediated 15.5% of the effect of substance abuse on suicide, though the indirect effect was not statistically significant, while the direct effect remained significant with a hazard ratio of 1.76, leading to a total effect hazard ratio of 1.90. These findings indicate that sleep disturbances is a more important mediator between substance abuse and suicide in PLHIV than depression (Figure 1).

## Discussion

In this study, 11 years of follow-up data were used to examine the association between substance abuse and suicide among PLHIV in Taiwan. Prior findings have suggested a link between substance abuse and suicide (Bernert et al., 2015; Devin et al., 2023), and numerous studies have explored the epidemiology of suicide among PLHIV with substance abuse. The findings of this study indicate PLHIV with substance abuse have a significantly (2.83) higher incidence of suicide than PLHIV without substance abuse (743.91 vs. 262.15 per 100,000 person-years, respectively). These results align with the findings from a study conducted in Iran (Dabaghzadeh et al., 2015) that also found substance abuse to be a risk factor for suicide among individuals living with HIV.

The heightened prevalence of substance abuse among PLHIV may be attributed to inflammation and immune cell activation resulting from virologically controlled HIV infection. This physiological response can disrupt sleep quality and circadian patterns through cytokine action (Baer et al., 2022; Faraut et al., 2018). In addition, living with a chronic illness such as HIV can induce stress and trigger sleep disturbances, exacerbating symptoms such as pain and depression and creating a cyclical pattern of substance abuse and heightened risk of suicidal behavior. Thus, the findings highlight the importance of addressing substance abuse as a crucial component in preventing sleep disturbances among individuals living with HIV (Dabaghzadeh et al., 2015; West et al., 2023).

Also, a significant association was identified in this study between substance abuse and low income, indicating an increased risk of suicide among PLHIV and a 1.93 times higher suicide incidence in PLHIV with substance abuse than their peers without. After adjusting for confounding factors, the hazard ratio for those in the low-income group was found to be 5.15 times higher than that for those in the high-income group. Increases in unemployment, economic growth, and lagged economic uncertainty have previously been linked to higher suicide risk (Mudiyanselage et al., 2025; Mudiyanselage, Tsai, Tsai, et al., 2024; Tsai et al., 2022). Also, stigma and discrimination can further undermine the ability of PLHIV to obtain and maintain employment, impacting various aspects of life, including access to treatment and care (Wang et al., 2019), underscoring the significance of addressing the broader socioeconomic determinants of health as part of suicide prevention efforts.

Prolonged sleep disturbance and depression increase the risk of substance abuse, and the rate of suicide due to overdose is estimated to be between 15% and 35% among PLHIV. Sleep disturbances and depression are prevalent symptoms among individuals with substance abuse and are significantly linked to higher risks of both suicidal ideation and suicidal behavior (Tsai et al., 2024). Substance abuse is also disproportionately prevalent among populations experiencing various forms of depression and sleep disturbances. The mediation analysis conducted in this study revealed that having a history of sleep disturbances accounted for a significant proportion of the mediation effect (19.1%) between substance abuse and suicide. However, the pathway effect analysis using potential outcomes demonstrated that sleep disturbances do mediate the impact of substance abuse on suicide. Substance abuse and depression are prevailing psychiatric disorders frequently linked to suicide (Mudiyanselage, Tsai, Tsai, et al., 2024; Tsai et al., 2023, 2024). In this study, depression accounted for only 15.5% of the mediation effect between substance abuse and suicide, while depression was found to have no significant mediating effect on this relationship (Tsai et al., 2024).

### Strengths and Limitations

This study has several notable strengths. First, using a population-based study with a highly representative sample of PLHIV in Taiwan between 2005 and 2016 minimizes the risk of selection bias. Second, using insurance claim data sets in clinical research allows access to longitudinal records for a large and diverse patient cohort. The ample data set size allowed for stratified analyses based on specific variables of interest, such as age and gender. The findings of this study identify elevated rates of suicidality among PLHIV with substance abuse and low income. In addition, the findings confirm that sleep disturbances play a mediating role in the association between substance abuse and suicide, with a statistically significant mediation effect. These findings contribute scientific evidence to support clinical practice and inform the development of protocols aimed at preventing suicidal behaviors and managing the well-being of individuals living with HIV and substance abuse worldwide.

This study was also affected by several important limitations. First, determining the suicide rates is a complex task due to challenges in distinguishing suicides from accidental deaths. In recent years, traffic accidents have been used as a method of suicide. Those who contemplate, attempt, or die by suicide often endure significant psychological distress and may perceive themselves as a burden to their loved ones, particularly in the context of mental disorders and stigma (Tsai et al., 2024). However, differentiating between suicides and traffic accidents can be challenging, as the true intent may be obscured by factors such as common reasons, timing, and locations associated with traffic accidents. Second, there is the potential for misclassification time bias in suicide cases due to our sole reliance on claims data. In this study, HIV population data and death registration data were used to ascertain the cause of death, which is typically determined by a physician based on clinical symptoms (Chen et al., 2021). In many Asian cultures, suicide is considered taboo and shameful, leading families to remain silent or conceal information to preserve their reputation, resulting in limited details being recorded on death certificates. Moreover, suicide is not typically covered by insurance claims, further diminishing the reliability of suicide-specific information (Katz et al., 2016). It is important to note that, while Taiwan’s death records data are generally regarded as reliable, underreporting and misclassification are more prevalent for suicides compared to other causes of death. Furthermore, our data indicate the number of female participants in substance abuse and suicide to be small. As a result, the observed gender differences are based on a limited sample size of female participants in suicide events. Further analysis with a larger sample will be necessary to validate these findings.

In the diagnostic process for substance abuse, it is imperative to include screening for sleep disturbances and suicidal behavior. However, further research is warranted to investigate the potential effects of treatment for substance abuse on suicide risk and the mental health of patients with substance abuse. In future studies, personal health factors, including sleep quality, HIV status disclosure, HIV staging, CD4 cell count, and viral load, as well as social support, quality of life, family history of mental illness, and family history of suicide, should be systematically collected for PLHIV with substance abuse who died by suicide.

### Conclusions

In this study, a large and representative cohort of 14,549 PLHIV was used and was followed longitudinally for 11 years. Over the period of study, 59 cases of suicide were identified. The findings indicate that the incidence of suicide is 1.93 times higher in PLHIV with substance abuse than in PLHIV without substance abuse, and that PLHIV with low income have a suicide incidence rate 5.15 times higher than those with high income. The results of the mediation analysis indicate that sleep disturbances mediate 19.1% of the effect between substance abuse and suicide, with a statistically significant direct relationship. However, no significant mediating effect was found for depression on the relationship between substance abuse and suicide. These findings provide substantial evidence to guide clinical practice and facilitate the development of protocols aimed at preventing suicidal behaviors and promoting well-being in PLHIV with substance abuse on a global scale.
